# Quantifying the Association between *Campylobacter* Infection and Guillain-Barré Syndrome: A Systematic Review

**DOI:** 10.3329/jhpn.v28i6.6602

**Published:** 2010-12

**Authors:** Kate O. Poropatich, Christa L. Fischer Walker, Robert E. Black

**Affiliations:** ^1^ George Washington School of Medicine and Health Sciences, Washington, DC, USA; ^2^ Department of International Health, Johns Hopkins Bloomberg School of Public Health, Baltimore, MD, USA

**Keywords:** *Campylobacter*, *Campylobacter* infections, Guillain-Barré Syndrome, Review literature

## Abstract

Guillain-Barré Syndrome (GBS) is a neurologic disease that causes ascending paralysis and is triggered by a preceding bacterial or viral infection. Several studies have shown that patients with GBS have a recent history of infection due to *Campylobacter jejuni*. A literature review of published studies that reported rates of *Campylobacter* infection before or in conjunction with GBS was done. These reported data were used for calculating the proportion of GBS cases who tested positive for *Campylobacter* compared to the control population and the incidence of GBS among patients infected with *Campylobacter*. Results of the analysis suggest that 31% of 2,502 GBS cases included in these papers are attributable to *Campylobacter* infection.

## INTRODUCTION

Since the near global eradication of poliomyelitis, Guillain-Barré Syndrome (GBS) has become the most common cause of acute neuromuscular paralysis (1). GBS is an acute neurologic disease driven by autoimmunity and molecular mimicry in which the body stages a cell-mediated and humoral immunological response against peripheral nerve myelin (2). Asbury and Cornblath clinically defined GBS as a progressive motor weakness of more than one limb with low or absent reflexes and no other identifiable cause (3). The global incidence of GBS ranges from 0.4 to 4.0 (median 1.3) cases per 100,000 people annually, occurring slightly more often in adolescents and young adults than in children (2, 3). In its acute phase, GBS can cause severe disability and even death (4). A recent systematic review of GBS estimated that 40–70% of all GBS cases are preceded by an acute infectious illness, of which 22–53% are upper respiratory infections and 6–26% are gastrointestinal infections, one of the most common being enteritis due to *Campylobacter* (5, 6).

GBS can be classified into demyelinating and axonal subtypes. The demyelinating subtype—acute inflammatory demyelinating polyneuropathy (AIDP)—is characterized by demyelination of neurons whereas demyelination is absent in the axonal subtypes—acute motor axonal neuropathy (AMAN) and acute motor sensory axonal neuropathy (AMSAN) (7). AIDP is common in North America and Europe while AMAN/AMSAN have been more commonly found in studies in China, Japan, and Mexico (2, 8, 9). Although both the subtypes have been associated with infection due to *Campylobacter*, it is has been shown that *Campylobacter* is more widely associated with AMAN (10–12).

*Campylobacter jejuni* was first associated with GBS in 1982 when Rhodes and Tattersfield reported a case of GBS following enteric infection with *C. jejuni* (13, 14). It is difficult to positively associate *C. jejuni* with GBS because the bacteria are usually eliminated from the body within 16 days of infection and before the onset of neurological symptoms, which normally begin 10 days to 3 weeks after the onset of diarrhoea (1, 2). Although *Campylobacter* is prevalent in most parts of the world, it is not yet routinely diagnosed in rural health clinics. For this reason, many *Campylobacter*-associated GBS cases may go unrecognized because by the time the person presents with GBS, *Campylobacter* is no longer present (15, 16).

Results of a previous overview of literature suggested that *Campylobacter* infection is responsible for 13–72% of GBS cases; however, this review was not designed to systematically review the literature (2). Thus, we did a systematic review of published studies to estimate the proportion of GBS cases that may be attributed to *Campylobacter* among persons of all ages and from all regions of the world.

## MATERIALS AND METHODS

We performed a PubMed search of studies published from July 1982 to 28 June 2010 that investigated the relationship between infection due to *Campylobacter* and GBS. We searched using combinations of the following Medical Subjects Headings (MeSH): ‘Guillain-Barré Syndrome’ and ‘campylobacter’ and the key words: ‘guillain barré syndrome’, ‘GBS’, ‘acute autoimmune neuropathy’, ‘acute inflammatory polyneuropathy’, ‘acute inflammatory demyelinating polyneuropathy’, ‘AIDP’, ‘AMAN’, and ‘campylobacter’. We also searched the reference lists of retrieved manuscripts to identify additional studies.

We included cohort studies of persons with laboratory-confirmed infection due to *Campylobacter* who were followed prospectively to assess subsequent GBS cases and retrospective case-control studies that tested for *Campylobacter* infections among GBS-confirmed cases and non-GBS controls. We excluded case-control studies with fewer than 15 GBS cases and cohort studies, including cases of GBS that developed more than six months after a confirmed infection due to *Campylobacter*. Studies were included if serum and stool samples were collected during the acute phase of GBS—within 24–48 hours of patient's admission to hospital and no longer than four weeks after admission. Studies were excluded if they relied on a complement fixation assay (CFA) for the diagnosis of *Campylobacter*.

For case-control studies, the primary outcome considered was laboratory-confirmed presence of infection due to *C. jejuni* in GBS cases and controls. For cohort studies, the primary outcome considered was the development of GBS in persons with laboratory-confirmed infection due to *Campylobacter*. Clinical features of GBS were analyzed, and antecedent infections were investigated. Definitions of GBS in the studies were based on currently-accepted criteria for diagnosing GBS (i.e. a progressive, symmetric ascending paralysis with a relative sensory sparing in more than one extremity with hypo- or areflexia) (3, 17). Studies were excluded from review if these did not explicitly state or cite their criteria for diagnosis of GBS. Studies were included if these used appropriate microbiological methods (serological assays and stool cultures) for detecting *Campylobacter* species (13).

We reviewed all titles and abstracts to identify eligible studies. Full manuscripts were obtained for potentially eligible studies.

### Statistical methods

For case-control studies, we calculated the median and interquartile range (IQR) for cases and controls and *Campylobacter*-positive cases and controls. The Microsoft Excel software was used for calculating medians and IQR (18).

## RESULTS

We screened 573 potential studies (Fig.) for inclusion in the review. After applying the inclusion and exclusion criteria, we included two prospective cohort studies (16, 19) and 30 case-control studies (Table). In total, case-control studies yielded 2,502 GBS subjects and 3,419 controls. One study took place in the UN-classified (20) least-developed countries (21)—11 in developing countries (10, 11, 22–30) and 20 in developed countries (12, 16, 19, 31–47).

**Fig.**
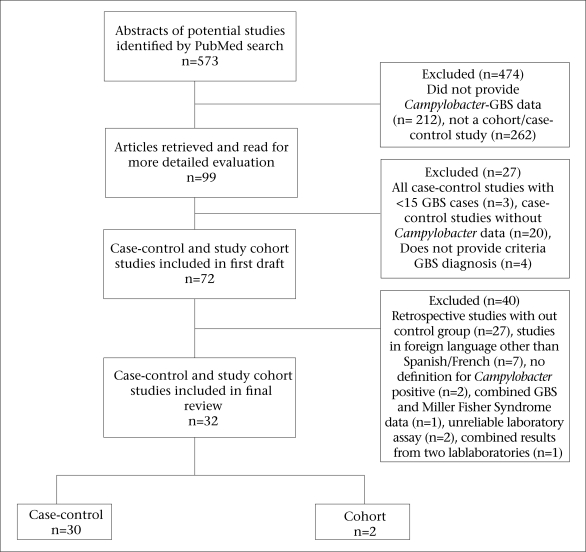
Results of literature review

**Table.** Proportions of *Campylobacter* infections in GBS case-control studies

Author	Year	Country	Duration of study	GBS cases			Controls	
				Study population with GBS	Positive for *Campylobacter* No. (%)	GBS cases with AMAN/AIDP (% positive for *Campylobacter*)	Controls**	Positive for Campylobacter** No. (%)
Islam	2010	Bangladesh	July 2006–June 2007	97	55 (56.7)*	14/64 AIDP (50.0) 43/64 Axonal†† (79.1)	97 HOC 97 OND	0 HOC 0 OND
Kalra	2009	India	2003–2006	54	15 (27.7)†		42 HC 43 OND	1 (2.3) HC 1 (2.3) OND
Wierzba	2008	Egypt	Apr 2001–Sep 2003	133	14 (10.5)†	76/120 AIDP 17/120 AMAN	374 HC	26 (7.0)
Nagashima	2007	Japan	Aug 1999–Feb 2004	73	23 (31.5)†		73 HC	2 (2.7)
Sinha	2007	India	Feb 2001–Mar 2005	80	21 (26.3)†	34 AIDP (5.9) 46 Axonal†† (41.3)	80 HC 45 OND	4 (5.0) HC 2 (4.4) OND
Gorthi	2006	India	Nov 1997–Aug 1998	20	7 (35.0)†	1 AIDP	20 HOC 20 OND	5 (25.0) HOC 0 OND
Schmidt-Ott	2006	Germany	1993–2003	36	23 (63.9)†		57 NC	2 (3.5)
Sinha	2004	India	Jun 2001–Mar 2003	42	2 (4.8)*	18 AIDP (5.6) 24 Axonal†† (29.2)	42 NC	0
Liu	2003	China		51	19 (37.3)†	20 AIDP (42.1) 28 AMAN (57.9)	51 NC	3 (5.9)
Ho	1999	China	Jan 1992–Dec 1997	138	99 (71.7)†	26 AIDP (50) 72 AMAN (81)	39 NC	6 (15.3)
Koga	1999	Japan		152	36 (23.7)†		78 OND 69 NC	2 (2.6) OND 6 (8.7) NC
Yuki	1999	China	Jun 1993–Oct 1995	55	26 (47.3)†	9 AIDP (0) 28 AMAN (53.6)	101 NC	5 (5.0)
Guarino	1998	Italy	1992–1993	55	8 (14.5)†		96 HC	3 (3.1)
Hao	1998	Japan	1990–1996	205	92 (44.9)†		100 NC	1 (1.0)
Koga	1998	Japan		201	62 (30.8)†		101 OND 46 NC	8 (8.0) OND 2 (4.3) NC
Jacobs	1998	Netherlands	Jun 1986–Dec 1989, Sep 1990–Sep 1992	154	49 (31.8)†		154 OND	18 (11.7)
Saida	1997	Japan	1990–1996	205	92 (44.9)†		100 NC	1 (1.0)
Jacobs	1996	Netherlands		154	46 (31.8)†		63 OND 50 NC	7 (11.1) OND 4 (8.0) NC
Ho	1995	China	Jan 1992–Oct 1992	38	25 (65.8)†	12 AIDP (41.7) 21 AMAN (76.2)	82 NC	13 (15.9)
Rees	1995	UK	Nov 1992–April 1994	96	25 (26.0)§		83 HC 94 HOC	1 (1.2) HC 2 (2.1) HOC
Tang	1995	China	July 1991–Nov 1993	16	8 (50)		90 NC 32 OND	4 (4.4) NC 9 (28.1) OND
Enders	1993	Germany	1988–1992	38	15 (39.5)†		39 NC 109 OND	7 (17.9) NC 8 (7.3)
Gregson	1993	UK	Jan 1987–Feb 1991	42	15 (35.7)†		41 NC	0
Yuan	1993	China	Jul 1991–Oct 1992	17	9 (52.9)†		17 OND 33 NC	3 (17.6) OND 3 (9.1) NC
Kuroki	1993	Japan	Feb 1985–Jan 1992	46	14 (30.4)*		503 NC	6 (1.2)
Mishu	1993	USA: MD, NJ, MO	1983–1990	118	43 (36.4)†		103 OND+NC	10 (9.7)
Vriesendorp	1993	USA	7 years	58	10 (17.2)†		42 OND 29 NC	3 (7.1) OND 2 (6.9) NC
Speed	1987	Australia		45	19 (42.2)†		12 NC	0
Speed	1984	Australia		27	9 (33.3)†		15 OND	1 (6.7)
Kaldor	1984	Australia	Jan 1979–Dec 1982	56	21 (37.5)†		27 OND 30 NC	0 OND 0 NC

*Stool culture;

†Serological (ELISA),

§Combined results;

AIDP=Acute inflammatory demyelinating polyneuropathy;

††AMAN=Acute motor axonal neuropathy;

**OND=Controls with other neurological disorders (non-GBS);

AMSAN=Acute motor sensory axonal neuropathy;

DC=Controls with other diarrhoea infections;

ELISA=Enzyme-linked immunosorbent assay;

GBS=Guillain-Barré Syndrome;

HC=Hospital controls;

HOC=Household controls;

MD=Maryland;

MO=Missouri;

NC=Normal/healthy controls;

UK=United Kingdom;

US=United States

For case-control studies, rates of *Campylobacter* infection varied among the GBS patients and controls from 4.8% to 71.7% and 0% to 28.1% respectively. The median positivity for *Campylobacter* among the GBS cases was 35.4% (IQR 28.3–44.9), and among the controls, it was 4.4% (IQR 1.2–8.8), suggesting that 31.0% of the GBS cases may be attributable to a previous infection due to *Campylobacter*.

We identified two cohort studies meeting our inclusion criteria (16, 19). In the study by McCarthy *et al.,* 0.03% of *Campylobacter* cases (n=29,563) developed GBS, which can be expressed as 30.4 cases of GBS per 100,000 cases of *C. jejuni*-associated infection [95% confidence interval (CI) 13.9–57.8] (16). In a population-based cohort study, Tam *et al.* found that, of 2,560 persons infected with *Campylobacter* who received subsequent medical follow-up, three cases of GBS were found, yielding an incidence of 117 per 100,000 cases of *C. jejuni* (95% CI 0.38–3.63) (19).

## DISCUSSION

In this review, we found 32 studies that met our inclusion/exclusion criteria and measured the association of infection due to *Campylobacter* with GBS. One of the challenges in determining the incidence of *Campylobacter*-associated GBS is that many cases of *Campylobacter* go unreported. For this reason, most studies focus on a sample of persons already diagnosed with GBS and perform retrospective analyses to determine if they were infected with *C. jejuni* (2). Large cohort studies are ideal for determining the actual incidence of *Campylobacter*-associated GBS because they identify *Campylobacter* cases at the time of active infection and are, thus, less likely to miss a *Campylobacter* infection because of poor timing. Unfortunately, these studies are rare. We used a standard definition for GBS to minimize differences in diagnostic criteria among studies (3). These are the internationally-accepted criteria currently used for diagnosing GBS and helped control for study heterogeneity.

One limitation of this analysis is controlling for heterogeneity among studies that met our inclusion criteria. Depending on whether studies used serologic assays or stool samples for the diagnosis of *Campylobacter*, different positive values could be shown. Serology is the preferred mechanism of detection because *Campylobacter*-specific antibodies can be detected in serum of the patient for an indefinite length of time compared to *Campylobacter* antigens in stool samples, which are cleared, on average, 16 days after infection. Thus, there could be many false-negative stool cultures in GBS patients in studies that rely solely on stool culture for the diagnosis of *Campylobacter* (48). Additionally, there is no serological test that is specific for *Campylobacter* infection as far back as in the two months before the onset of GBS; so, there is potential misclassification of exposure in retrospective serological studies, which comprise the bulk of the case-control studies considered in this analysis.

Variations in serological assays could also affect results of study; antigens used and endpoints for positivity often vary with different assays (48). It is preferable that studies using serology to adhere to strict criteria for recent diagnosis of *Campylobacter* (i.e. positive ELISA for at least 2 classes of antibodies) to avoid false-positive results. However, we did not specifically exclude studies on the basis of the number of *Campylobacter*-specific antibodies they used, only if they did not explicitly state what their standard was for seropositivity of *Campylobacter*. Finally, we did not control for improving technologies for the detection of *Campylobacter* over the years of the studies, which could also confound the comparison of results from older studies with more recent studies.

Another limitation to take into consideration is the overestimation of *Campylobacter* infectivity in control groups. While serological evidence of *Campylobacter* infection is likely indicative of recent infection in GBS cases due to the strong temporal link between *Campylobacter* infection and GBS, this is not the case for controls. If controls test positive, there is no way of knowing how long ago their infection occurred because antibody titres remain elevated for an extended period. The median value of 4.4% for *Campylobacter* positivity in the control group in our study may represent individuals with infection due to *Campylobacter* beyond two months and, thus, could be an overestimate of active *Campylobacter* infections in the general population. If this were the case, the prevalence of *Campylobacter*-associated infections would actually be greater between cases and controls.

The instance of heterogeneous control populations in the studies is an additional limitation of this analysis. Some studies included controls with other neurological disorders with preceding *C. jejuni*-associated infection as high as 28.1%, which is considerably higher than that has been observed in other control groups. Selection of controls in the study could alter the difference in *Campylobacter* positivity between GBS cases and controls, depending on whether the control groups' risk of exposure was the same as the GBS group or different. For instance, household controls could have a risk comparable to GBS cases, and high rates of seropositivity among these controls could reflect transmission of *Campylobacter* from cases and controls, along with unhygienic living conditions in developing countries.

We were also unable to search the Chinese medical literature databases or review Chinese language papers identified in PubMed. We recognize that these data may exist and would better help us understand the associations of GBS with *Campylobacter* in China.

While infection due to *Campylobacter* is not normally associated with high rates of mortality in developed countries, 4–15% of patients with GBS may die within the first year after onset (49). *C. jejuni* has been identified as a potential predictor of poor outcome in persons suffering from GBS for inducing a more severe autoimmune response and greater axonal damage (2). Some studies report that preceding *Campylobacter* infection can increase the severity of GBS in patients, i.e. death, mechanical ventilation, etc. (2). This is problematic in poorer countries that usually have increased frequencies of infection due to *Campylobacter* because persons who develop GBS may have limited access to healthcare and treatment required for GBS. For these reasons, the appropriate measures must be taken to reduce the incidence of *Campylobacter*-associated GBS. This can be achieved through reducing the frequency of *Campylobacter* cases by improving sanitation, preventing the faecal-oral routes of transmission of *Campylobacter*.

## References

[1] Allos BM (1997). Association between *Campylobacter* infection and Guillain-Barré syndrome. J Infect Dis.

[2] Hadden RD, Gregson NA (2001). Guillain-Barré syndrome and *Campylobacter jejuni* infection. Symp Ser Soc Appl Microbiol.

[3] Asbury AK, Cornblath DR (1990). Assessment of current diagnostic criteria for Guillain-Barré syndrome. Ann Neurol.

[4] Korinthenberg R, Mönting JS (1996). Natural history and treatment effects in Guillain-Barré syndrome: a multicentre study. Arch Dis Child.

[5] McGrogan A, Madle GC, Seaman HE, de Vries CS (2009). The epidemiology of Guillain-Barré syndrome worldwide. A systematic literature review. Neuroepidemiology.

[6] Jacobs BC, Van Belkum A, Endtz HP, Nachamkin I, Szymanski CM, Blaser MJ (1998). Guillain-Barré syndrome and *Campylobacter* infection. Campylobacter, 3rd ed..

[7] Hafer-Macko C, Hsieh ST, Li CY, Ho TW, Sheikh K, Cornblath DR (1996). Acute motor axonal neuropathy: an antibody-mediated attack on axolemma. Ann Neurol.

[8] Paradiso G, Tripoli J, Galicchio S, Fejerman N (1999). Epidemiological, clinical, and electrodiagnostic findings in childhood Guillain-Barré syndrome: a reappraisal. Ann Neurol.

[9] Ogawara K, Kuwabara S, Mori M, Hattori T, Koga M, Yuki N (2000). Axonal Guillain-Barré syndrome: relation to anti-ganglioside antibodies and *Campylobacter jejuni* infection in Japan. Ann Neurol.

[10] Ho TW, Willison HJ, Nachamkin I, Li CY, Veitch J, Ung H (1999). Anti-GD1a antibody is associated with axonal but not demyelinating forms of Guillain-Barré syndrome. Ann Neurol.

[11] Ho TW, Mishu B, Li CY, Gao CY, Cornblath DR, Griffin JW (1995). Guillain-Barré syndrome in northern China. Relationship to *Campylobacter jejuni* infection and anti-glycolipid antibodies. Brain.

[12] Vriesendorp FJ, Mishu B, Blaser MJ, Koski CL (1993). Serum antibodies to GM1, GD1b, peripheral nerve myelin, and *Campylobacter jejuni* in patients with Guillain-Barré syndrome and controls: correlation and prognosis. Ann Neurol.

[13] Prendergast MM, Moran AP (2000). Lipopolysaccharides in the development of the Guillain-Barré syndrome and Miller Fisher syndrome forms of acute inflammatory peripheral neuropathies. J Endotoxin Res.

[14] Rhodes KM, Tattersfield AE (1982). Guillain-Barré syndrome associated with *Campylobacter* infection. Br Med J (Clin Res Ed).

[15] Hughes RA, Hadden RD, Gregson NA, Smith KJ (1999). Pathogenesis of Guillain-Barré syndrome. J Neuroimmunol.

[16] McCarthy N, Giesecke J (2000). Incidence of Guillain-Barré syndrome following infection with *Campylobacter jejuni*. Am J Epidemiol.

[17] Criteria for diagnosis of Guillain-Barré syndrome (1978). Ann Neurol.

[18] Microsoft Excel (2008). Microsoft Excel 2008 for MAC. Version 12.1.0.

[19] Tam CC, Rodrigues CL, Peterson I, Islam A, Hayward A, O'Brien JO (2006). Incidence of Guillain-Barré syndrome among patients with *Campylobacter* infection: a general practice research database study. J Infect Dis.

[20] United Nations Statistics Division (2010). Composition of macro geographical (continental) regions, geographical sub-regions, and selected economic and other groupings.

[21] Islam Z, Jacobs BC, van Belkum A, Mohammad QD, Islam MB, Herbrink P (2010). Axonal variant of Guillain-Barré syndrome associated with *Campylobacter* infection in Bangladesh. Neurology.

[22] Kalra V, Sankhyan N, Sharma S, Gulati S, Choudhry R, Dhawan B (2009). Outcome in childhood Guillain-Barré syndrome. Indian J Pediatr.

[23] Wierzba TF, Abdel-Messih IA, Gharib B, Baqar S, Hendaui A, Khalil I (2008). *Campylobacter* infection as a trigger for Guillain-Barré syndrome in Egypt. PLoS One.

[24] Sinha S, Prasad KN, Jain D, Pandey CM, Jha S, Pradhan S (2007). Preceding infections and anti-ganglioside antibodies in patients with Guillain-Barré syndrome: a single centre prospective case-control study. Clin Microbiol Infect.

[25] Gorthi SP, Kapoor L, Chaudhry R, Sharma N, Perez-Perez GI, Panigrahi P (2006). Guillain-Barré syndrome: association with *Campylobacter jejuni**Mycoplasma pneumoniae* infections in India. Natl Med J India.

[26] Sinha S, Prasad KN, Pradhan S, Jain D, Jha S (2004). Detection of preceding *Campylobacter jejuni* infection by polymerase chain reaction in patients with Guillain-Barré syndrome. Trans R Soc Trop Med Hyg.

[27] Liu GF, Wu ZL, Wu HS, Wang QY, Zhao-Ri GT, Wang CY (2003). A case-control study on children with Guillain-Barré syndrome in north China. Biomed Environ Sci.

[28] Yuki N, Ho TW, Tagawa Y, Koga M, Li CY, Hirata K (1999). Autoantibodies to GM1b and GalNAc-GD1a: relationship to *Campylobacter jejuni* infection and acute motor axonal neuropathy in China. J Neurol Sci.

[29] Tang J, Yuan J, Hao H (1995). GM1 antibody in Guillain-Barré syndrome after *Campylobacter jejuni* infection. Chin Med J.

[30] Yuan JM, Tang J, Chen QT, Xiao L, Hao HJ, Jia ZD (1993). Guillain-Barré syndrome and *Campylobacter jejuni* infection. A study on the etiological characteristics of Guillain-Barré syndrome in China. Chin Med J.

[31] Nagashima T, Koga M, Odaka M, Hirata K, Yuki N (2007). Continuous spectrum of pharyngeal-cervical-brachial variant of Guillain-Barré syndrome. Arch Neurol.

[32] Schmidt-Ott R, Schmidt H, Feldmann S, Brass F, Krone B, Gross U (2006). Improved serological diagnosis stresses the major role of *Campylobacter jejuni* in triggering Guillain-Barré syndrome. Clin Vaccine Immunol.

[33] Koga M, Yuki N, Hirata K (1999). Subclass distribution and the secretory component of serum IgA anti-ganglioside antibodies in Guillain-Barré syndrome after *Campylobacter jejuni* enteritis. J Neuroimmunol.

[34] Guarino M, Casmiro M, D'Alessandro R (1998). *Campylobacter jejuni* infection and Guillain-Barré syndrome: a case-control study. Emilia-Romagna Study Group on Clinical and Epidemiological Problems in neurology. Neuroepidemiology.

[35] Hao Q, Saida T, Kuroki S, Nishimura M, Nukina M, Obayashi H (1998). Antibodies to gangliosides and galactocerebroside in patients with Guillain-Barré syndrome with preceding *Campylobacter jejuni* and other identified infections. J Neuroimmunol.

[36] Koga M, Yuki N, Takahashi M, Saito K, Hirata K (1998). Close association of IgA anti-ganglioside antibodies with antecedent *Campylobacter jejuni* infection in Guillain-Barré and Fisher's syndromes. J Neuroimmunol.

[37] Jacobs BC, Rothbarth PH, van der Meché FG, Herbrink P, Schmitz PI, de Klerk MA (1998). The spectrum of antecedent infections in Guillain-Barré syndrome: a case-control study. Neurology.

[38] Saida T, Kuroki S, Hao Q, Nishimura M, Nukina M, Obayashi H (1997). *Campylobacter jejuni* isolates from Japanese patients with Guillain-Barré syndrome. J Infect Dis.

[39] Jacobs BC, van Doorn PA, Schmitz PI, Tio-Gillen AP, Herbrink P, Visser LH (1996). *Campylobacter jejuni* infections and anti-GM1 antibodies in Guillain-Barré syndrome. Ann Neurol.

[40] Rees JH, Gregson NA, Hughes RA (1995). Anti-ganglioside GM1 antibodies in Guillain-Barré syndrome and their relationship to *Campylobacter jejuni* infection. Ann Neurol.

[41] Enders U, Karch H, Toyka KV, Michels M, Zielasek J, Pette M (1993). The spectrum of immune responses to *Campylobacter jejuni* and glycoconjugates in Guillain-Barré syndrome and in other neuroimmunological disorders. Ann Neurol.

[42] Gregson NA, Koblar S, Hughes RA (1993). Antibodies to gangliosides in Guillain-Barré syndrome: specificity and relationship to clinical features. Q J Med.

[43] Kuroki S, Saida T, Nukina M, Haruta T, Yoshioka M, Kobayashi Y (1993). *Campylobacter jejuni* strains from patients with Guillain-Barré syndrome belong mostly to Penner serogroup 19 and contain beta-N-acetylglucosamine residues. Ann Neurol.

[44] Mishu B, Ilyas AA, Koski CL, Vriesendorp F, Cook SD, Mithen FA (1993). Serologic evidence of previous *Campylobacter jejuni* infection in patients with the Guillain-Barré syndrome. Ann Intern Med.

[45] Speed BR, Kaldor J, Watson J, Newton-John H, Tee W, Noonan D (1987). *Campylobacter jejuni*/*Campylobacter* coli-associated Guillain-Barré syndrome. Immunoblot confirmation of the serological response. Med J Aust.

[46] Speed B, Kaldor J, Cavanagh P (1984). Guillain-Barré syndrome associated with *Campylobacter jejuni* enteritis. J Infect Dis.

[47] Kaldor J, Speed BR (1984). Guillain-Barré syndrome and *Campylobacter jejuni*: a serological study. Br Med J (Clin Res Ed).

[48] Nachamkin I, Allos BM, Ho T (1998). *Campylobacter* species and Guillain-Barré syndrome. Clin Microbiol Rev.

[49] Vucic S, Kiernan MC, Cornblath DR (2009). Guillain-Barré syndrome: an update. J Clin Neurosci.

